# An Experimental Test of Insect-Mediated Colonisation of Damaged *Pinus radiata* Trees by Sapstain Fungi

**DOI:** 10.1371/journal.pone.0055692

**Published:** 2013-02-06

**Authors:** James K. McCarthy, Eckehard G. Brockerhoff, Raphael K. Didham

**Affiliations:** 1 Scion (New Zealand Forest Research Institute), Christchurch, New Zealand; 2 School of Biological Sciences, University of Canterbury, Christchurch, New Zealand; 3 School of Animal Biology, University of Western Australia, Perth, Australia; 4 CSIRO Ecosystem Services, Centre for Environment and Life Sciences, Perth, Australia; Universidad Pública de Navarra, Spain

## Abstract

Vector-pathogen dynamics play a central role in understanding tree health and forest dynamics. There is substantial evidence that bark beetles act as spore vectors for many species of fungi that cause ‘sapstain’ discolouration of damaged trees and timber. However, the direct quantitative link between vector-mediated spore dispersal and subsequent sapstain colonisation of wood is not fully understood. Here, we used caged versus uncaged experimental logs to test whether the exclusion of bark beetles quantitatively alters the distribution and intensity of sapstain fungal spread within damaged trees. Using generalised linear mixed models, we tested the effect of bark beetle exclusion on sapstain intensity within and among cut logs at two plantation forest sites. Overall, sapstain was found on all logs regardless of caging treatment, indicating that sapstain colonisation can occur (to some degree) without arthropod vectors, probably via wind, rain-splash and, potentially, latent endophytic development. This was supported by the dominance of *Diplodia pinea* in fungal isolations taken from trees felled at the site, as this fungal species is known to disperse independently of bark beetles. However, the intensity of sapstain within and among experimental logs was significantly greater in uncaged than in caged logs, where beetle colonisation was significantly greater. This appeared to be driven by a significant within-log association between the intensity of staining and the intensity of beetle, and other arthropod, tunnelling and feeding activities. Taken together, these results strongly suggest that the dominant mechanism underlying the role of bark beetles in sapstain development in this study system is not vector-mediated spore dispersal, per se, but rather the facilitation of spore entry and hyphal development through tunnelling and feeding activities. We discuss the implications of these findings for forest management and the effective salvage-harvest of trees damaged by stochastic climate events such as storm and fire damage.

## Introduction

Bark beetles that colonise conifers are known to be associated with specific fungal species that cause sapstain. Sapstain is the discolouration of wood caused by darkly-pigmented fungal hyphae [1], [2] growing through the sapwood and water-conducting cells of susceptible live trees and fallen timber [3]. Sapstain fungi can increase tree mortality rates and reduce the cosmetic quality of the wood at harvest, and is of concern to forest managers worldwide. Sapstain is generally caused by three groups of fungi; the ophiostomatoid fungi (such as species of *Ceratocystis*, *Ceratocystiopsis* and *Grosmannia* [4], [5]), black yeasts such as *Hormonema dematioides* Lagerb. & Melin and *Aureobasidium pullulans* (de Bary) G. Arnaud, and dark moulds such as *Cladosporium* spp. and *Diplodia pinea* (Desm.) J. Kickx f. (syn. *Sphaeropsis sapinea* (Fr.) Dyko & B. Sutton) [6]. Sapstain discolouration is primarily an issue in dead or cut/storm damaged trees (hereafter referred to as “damaged”), because sapstain fungi require aerobic conditions for development, a state that rarely occurs under the high sapwood moisture content of healthy trees [6], [7]. Consequently, damaged trees are at high risk of sapstain fungal colonisation, which can occur within as little as five days after damage given optimal conditions for fungal growth [8]. The rapid rate of sapstain onset is often thought to be due, in large part, to the assisted dispersal of sapstain fungi by highly vagile bark beetle vectors [9], [10].

A large body of literature has identified and described the associations between bark beetles and sapstain (primarily ophiostomatoid) fungi in many regions of the world [11], [12], [13]. In some studies, correlative evidence has shown that the intensity of sapstain in dead and damaged trees is higher in the presence of bark beetles, whereas stain is rare in their absence [14]. However, in other regions of the world the most important stain-causing fungi can be non-vectored species such as *D. pinea* [15], [16], in which the main method of dispersal is abiotic (through wind, rain splash, and horizontal transmission via spores and mycelium from mature to young trees [17], [18], [19], [20]) and there is little evidence of active transport by bark beetles [21], [22], [23]. Nevertheless, even abiotically-dispersed species such as *D. pinea* almost certainly benefit from bark beetle attack on trees because direct fungal penetration of host tissue requires fresh wounds of the tree to allow spore entry [24]. This raises the question of whether the apparent association between beetle activity and sapstain development in damaged trees should necessarily be viewed as facilitation via vector-mediated spore colonisation, or as facilitation via vector-mediated hyphal spread within the timber.

In an ecological context, increases in the frequency of extreme weather events, causing severe windthrow [15] and promoting bark beetle outbreaks [25], are predicted to alter the “disease triangle” between three crucial factors affecting host-plant damage – plant pathogen, plant host, and environmental change [26], [27]. In the case of sapstain, this complexity is potentially exacerbated by significant variation in a fourth factor, pathogen-vector dynamics, driven by variation in bark beetle abundance and propensity to attack susceptible trees under differing environmental conditions. Although sapstain colonisation of wind-thrown trees is a secondary cause of tree damage (following damage from the storm itself), the complexities of vector-pathogen and pathogen-host relationships are nevertheless central to improved understanding of the onset and development of tree damage overall.

In an applied context, there is growing concern about the uncertainty regarding timeframes available to salvage harvest trees following storm- or fire-damage in production forests [15], [28], before their commercial value is reduced by sapstain. This is becoming increasingly important as climate patterns are changing and extreme weather events are becoming more frequent [29]. The interactions between environmental conditions, the composition of the complex of sapstain-causing species, and the potential for rapid colonisation of damaged timber by fungi are all thought to be important in harvest management decisions. However, few studies have attempted to directly test the degree to which bark beetle presence and attack rates causally determine the spatial extent and distribution of sapstain [15], [28], [30]. Knowledge of the role of bark beetles and their relative importance to the spread and extent of sapstain in damaged logs would provide decision-makers with tools to act appropriately in the face of insect attack on their damaged resource.

This study aims to experimentally test whether colonisation by bark beetles quantitatively increases the extent of sapstain fungi in logs through direct facilitation of spore dispersal, or primarily through their tunnelling and feeding activities that facilitate hyphal development of fungi that spread through abiotic dispersal. Our goal was to experimentally exclude the abundant non-native bark beetles *Hylurgus ligniperda* (F.) and *Hylastes ater* (Paykull) from experimental cut logs in *Pinus radiata* D. Don plantations in New Zealand, and compare the intensity and spatial distribution of sapstain colonisation within and among caged versus uncaged logs. Both of these abundant bark beetle species were accidentally introduced to New Zealand, and are now found throughout New Zealand wherever *P. radiata* forests occur [31], [32]. We find clear evidence that bark beetles facilitate sapstain distribution and intensity primarily through their tunnelling and feeding activities in this system, and not through facilitation of spore dispersal.

## Materials and Methods

### Site selection and experimental setup

Ten uncaged and ten caged logs (ca. 0.5 m in length) were placed at each of two forest stands in the Nelson region of the South Island, New Zealand. Nelson Forests Ltd provided private land access, and all necessary permits were obtained for the described field studies, which did not involve or affect any endangered or protected species. The 40 logs of 9–16 cm diameter (mean ± standard error: 12.1±0.3 cm), plus an additional six logs of the same size that were used to monitor sapstain development at regular intervals, were cut from four seven year-old *P. radiata* trees felled on the day the experiment was set up, on the 25th of January 2011. Apparently healthy trees suitable for bark beetle colonisation were selected, felled with a chainsaw, de-limbed and cut into ca. 0.5 m lengths in the early morning when bark beetle activity is low [33]. The 46 logs were checked to ensure there was no damage to the bark, and immediately enclosed inside a vehicle to prevent arthropod colonisation prior to caging. The period of time between cutting the logs and setting up the experiment did not exceed one day, and care was taken not to damage the logs in transit.

The two sites were selected in second-rotation *P. radiata* forests that had been harvested within the six months leading up to January 2011. Recently harvested sites were selected to ensure sufficient bark beetle activity was present to provide high colonisation rates of uncaged logs. Both sites were flat, un-shaded, and not flood-prone so that pooling of water did not affect the progression of beetle colonisation and stain within the logs.

Half of the logs were protected from bark beetle colonisation (caged) with aluminium mesh of 1.8×1.4 mm mesh-size that was small enough to exclude the common pine-infesting bark beetles present in New Zealand. Although rarely considered in sapstain studies, many smaller arthropods other than bark beetles might also colonise logs, and it is possible that their feeding and tunnelling activities might also influence fungal growth and distribution. Therefore, evidence of any colonisation by arthropods other than bark beetles was recorded and considered in the analyses.

As *H. ater* and *H. ligniperda* are known to preferentially colonise logs that are in contact with the ground [34], the full length of every log was placed in ground contact. At each site, the 10 uncaged and 10 caged logs were placed at random points in a 4.5×6 m grid pattern, all with the long axis of the log facing north, separated by an equal distance of 1.5 m from any other log. In addition, three sapstain monitoring logs were placed 1.5 m away from the experimental logs at each of the two sites. One of these three logs was cut open every two weeks, at each site, as a rough guide to visually monitor beetle colonisation and sapstain growth, in order to ensure we sampled at a suitable time when sufficient amounts of staining had occurred.

In order to identify the fungal species causing sapstain in these logs, three entire *P. radiata* trees, independent of the experimental and monitoring logs, were felled in nearby forest stands (several hundred metres from the experimental logs) at each of our study sites, and subsequently analysed to identify the species causing stain. Trees were of a similar diameter to the experimental logs, and were located a sufficient distance away so that beetle or sapstain colonisation did not directly influence the experiment, but close enough to be subject to similar biotic and abiotic conditions as the experimental logs. These separate trees were used for fungal isolation so that the fungi could be isolated immediately at the time of collection, rather than after the extended period required to process the experimental logs, during which the chance of successfully isolating the stain fungi would be reduced due to contamination and rapid drying.

### Log processing

The logs were left out for a total of 34 days during the warmest months of the southern hemisphere summer, when the prevalence of fungal staining is known to be at its peak [35]. The logs were collected when approximately half of each cut face in the monitoring logs was covered in stain. Each experimental log was removed from its cage, where necessary, and placed in sealed plastic bags, with the upper surface of the log precisely marked so that it was known later which surface was in contact with the ground. To limit post-collection stain growth, logs were kept as cool as possible by ensuring they were left in a shaded and cool area until processing, which was completed within four days of collection.

**Figure 1:**
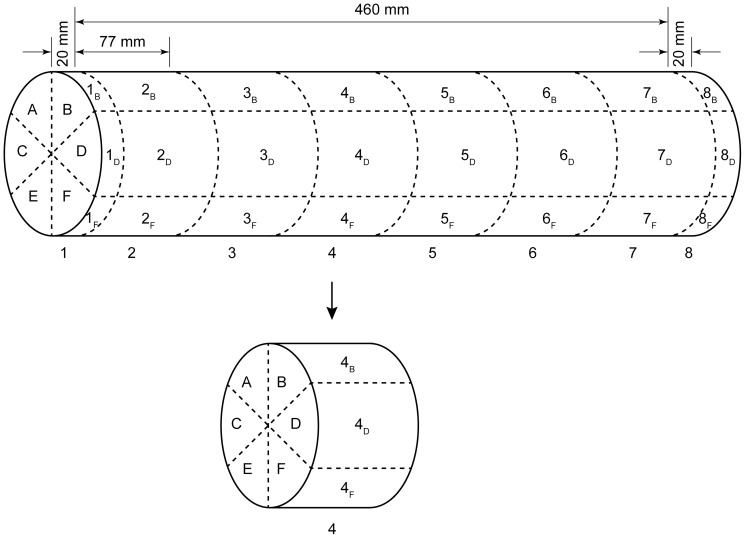
Schematic illustration of sampling areas on the experimental logs. All logs were assessed for bark beetle, other arthropod and sapstain colonisation. Numbers 1 to 8 indicate log sections (cuts are the dashed lines dividing the sections), and letters A to F indicate log segments within each section of the experimental *Pinus radiata* logs. The two outer sections, where sapstain penetration was expected to be greatest, were cut 20 mm thick, and the remaining length of log was divided into six equal-sized sections (ideally 77 mm, but varying from 75–79 mm, depending on variability between individual logs and individual section-cuts). The entire outer surface of the log was divided into discrete areas (1_A_ to 8_F_) where arthropod colonisation was recorded.

Measurements were taken of log length, diameter, and bark thickness at the ends and middle of each log. Each log was delineated into eight sections along the length of the log, and within each section, six radial segments were delineated, as illustrated in Figure 1. The two end sections (1 and 8) were thinner than the other sections in order to measure the amount of stain and beetle colonisation in the immediate vicinity of the exposed cut ends. The remaining six sections were divided equally into the remaining length of the log. As each log was around 0.5 m long, sections 2–7 were typically about 75 mm thick (±5 mm). Within each section, the radial segments closest to the ground had finer divisions (45°, as opposed to 90°) to increase the resolution of sapstain and arthropod counts where colonisation was expected to be greatest.

Logs were processed by first making a visual inspection of external arthropod colonisation in the external sectors indicated in Figure 1. To do this, the bark was carefully stripped away and bark beetles, their larvae, and their galleries were identified within each sector. Arthropod larvae and galleries that were not those of bark beetles were also counted, but not identified further. Following this, the logs were cut into the eight delineated sections, and a high-resolution photograph was taken of each cut face. Care was taken to ensure that the segments were aligned in their correct vertical orientation in the photo, allowing accurate placement of a digital grid over each image when making stain measurements. Note, that as there were eight sections this results in seven cut surfaces where stain was measured; these were between sections 1 and 2, 2 and 3, and so on, through to the cut face of segments 7 and 8. Only one image was required from one of the cut faces at each cut as the amount of stain on each face was identical to the other. The measurement points were then referred to as ‘section cuts’.

**Figure 2:**
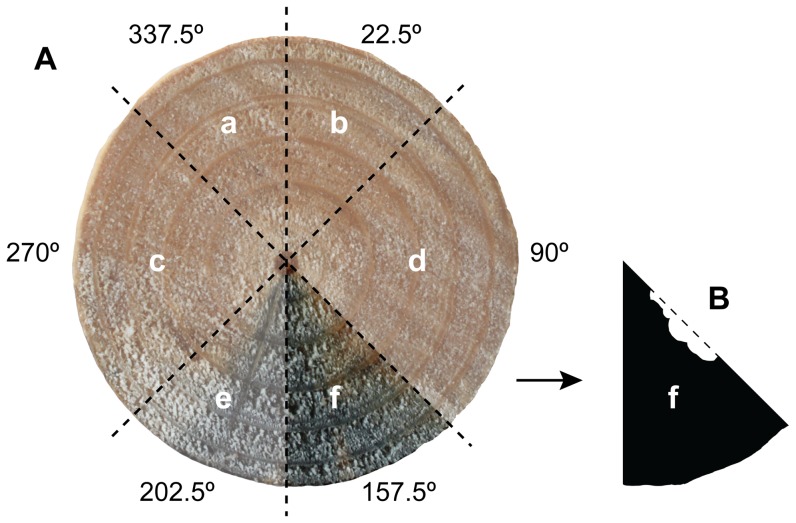
An example of the method used to calculate proportion sapstain cover. All cut faces (sections) had their proportion of stain cover digitally calculated. (A) shows the entire face of one *Pinus radiata* cut log section divided into six segments (a–f), and (B) shows a single segment extracted and digitally converted to a bitmap of relative colour intensity, in order to determine proportion sapstain coverage (in this case 0.944 cover of a 214151.04 pixel segment).

In order to measure the proportion stain in each segment, the entire image was imported into Adobe Photoshop® CS4 Extended, Version 11.0 (Adobe Systems Inc., San Jose, California, U.S.A.), and a radial grid was overlaid on the image, as illustrated in Figure 2. Each segment was extracted and saved as a single-coloured, black, bitmap image (one image file per segment) to determine total segment area. This process was then repeated for each segment, but this time manually drawing around stain-covered areas only. Image J software version 1.43u [36] was then used to calculate the number of pixels in the stain-covered area relative to the total segment area (Figure 2).

### Fungal isolation and identification

At the time of log collection, two 3 cm discs were cut from each of the three trees felled at both sites near the experimental logs. The first disc was taken from the cut end of the tree where the sapwood was exposed, and the second from 4.5 m up the length of the tree (to encourage sapstain by airborne fungal spores at the cut end, and beetle dispersed and endophytic fungal spores at the bark covered end). The discs were immediately labelled and sealed in plastic bags, and within 12 hours were moved to a cold room at 4°C in order to minimise moisture loss and slow fungal growth before isolations were taken.

Fungal isolation to identify and quantify representation of sapstain fungal species was undertaken within two days of sampling. All isolations were attempted on two agar media, one general malt medium for the isolation of most stain fungi present, and one selective medium with the addition of the eukaryote antibiotic cycloheximide, which ophiostomatoid fungi can tolerate [37]. The standard medium consisted of malt agar (3% malt extract, and 2% agarose) with 100 µg/ml streptomycin to inhibit bacterial growth, while the selective medium was the same as the standard but with 400 µg/ml cycloheximide added.

A wedge was taken from an arbitrary point within each disc using a hatchet. The wedge was bisected along the radial longitudinal plane using the hatchet and a mallet to initiate the split, and then separated manually, avoiding any external contact of the newly exposed surfaces. Five small chips were cut along the radial line from the newly exposed face using a sterile scalpel (as in McCarthy et al. 2012 [35]), the 1st and 5th of which were directly below the cambium, and above the pith, respectively. The remaining three were taken at equidistant lengths between the 1st and 5th chips. This was repeated twice per wedge, once inoculating the standard medium, and once inoculating the ophiostomatoid selective medium. After incubation periods of up to 10 weeks, emerging isolates were sub-cultured to tubes of malt agar (2% malt extract, and 2% agar). Bacterial colonies were recorded, but not isolated or identified further. Emerging fungal isolates were sorted into groups, and identified from their vegetative morphology and fruiting structures.

### Data analysis

The effect of bark beetle exclusion on variation in proportion sapstain cover was tested using a generalised linear mixed effects model (GLMM) with fixed categorical factors for the caging treatment, and log segment nested within log section, as well as random factors specified for site replicate and log replicate. The sapstain model was tested as a GLMM with binomial errors using the lme4 package in the R programming environment [38]. If overdispersion was evident in the fitting of the GLMM, then this was controlled for using a model with Poisson lognormal error structure [39]. Model simplification was performed using an information theoretic approach with AICc (Akaike Information Criterion corrected for small sample size) and Akaike weight (W_m_) to rank and subsequently select the best model describing the data, as recommended by Burnham & Anderson [40].

To assess the effect of the caging treatment, evidence of arthropod colonisation was separated into a binary ‘beetle evidence’ dataset (evidence = 1, no evidence = 0), where bark beetle evidence constituted the presence of adults, larvae, or galleries, and a binary ‘other arthropod evidence’ dataset, where the evidence of arthropods other than bark beetles constituted the presence of the arthropod larvae themselves or their galleries. For each sapstain measurement, corresponding measures of beetle evidence and other arthropod evidence were recorded from the two adjacent segments (combined) on either side of the cut-face (e.g. 2_B_ and 3_B_, Figure 1).

Finally, to test the relative contribution of bark beetle colonisation and colonisation by other arthropods in explaining the distribution of sapstain within caged and uncaged logs, the sapstain GLMM was repeated with ‘beetle evidence’ and ‘other arthropod evidence’ entered as covariate predictors ahead of the fixed caging, section and segment variables in the model. Models were compared and selected using AICc and W_m_ values, as above. If the spatial distribution of beetle and/or other arthropod colonisation was sufficient in its own right to explain the spatial distribution of sapstain then the covariate would subsume all the variance attributable to the fixed factors in the model. All statistical analyses were performed using R 2.13.1 software [41].

## Results

### Spatial distribution of sapstain

**Figure 3:**
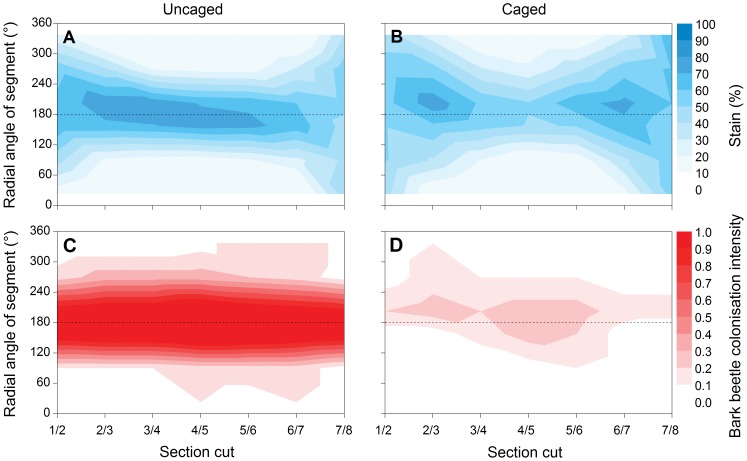
Beetle colonisation and sapstain development in caged versus uncaged logs. Comparison of average sapstain intensity for (A) uncaged and (B) caged *Pinus radiata* experimental logs, as well as the corresponding bark beetle (*Hylastes ater* and *Hylurgus ligniperda*) colonisation intensity for the same (C) uncaged and (D) caged logs (n = 20 experimental logs in each case). The radial angle of the segment indicates the spatial orientation of the log, with 180° representing the point of ground contact, as indicated by the dashed line. Bark beetle attack intensity is measured as the frequency of attack across logs.

**Figure 4:**
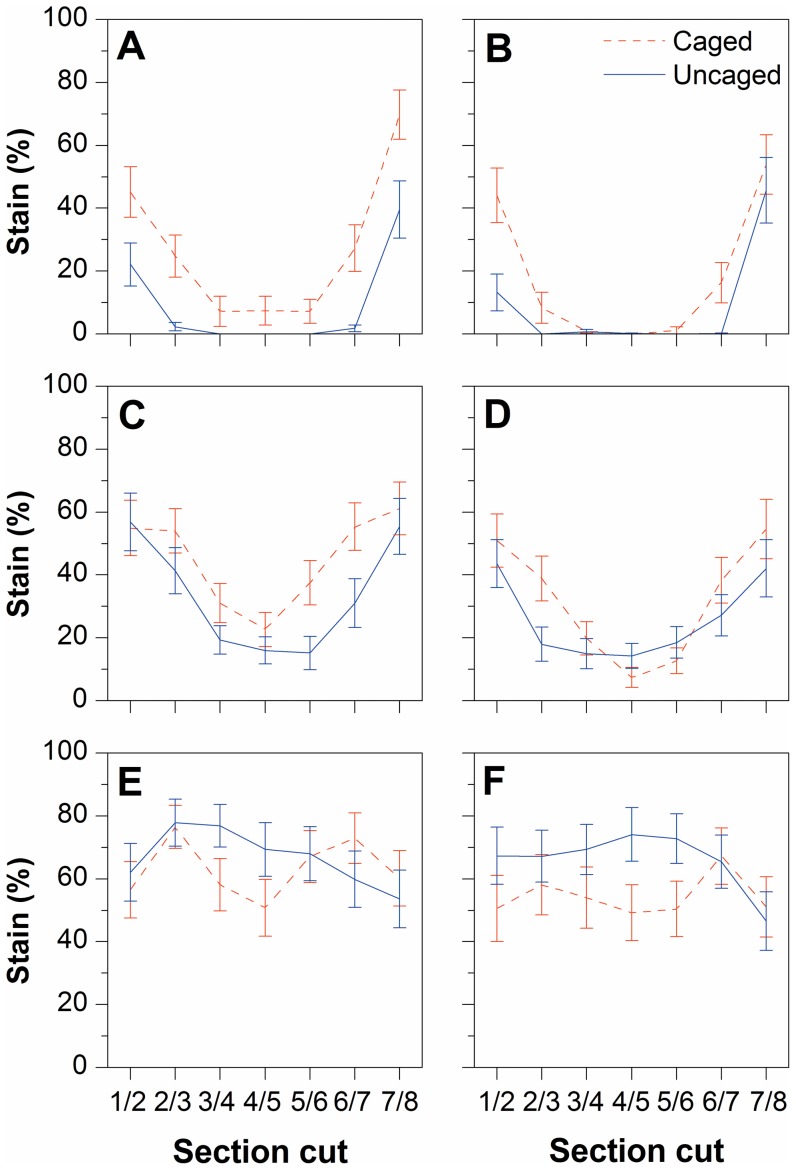
Comparison of stain distribution per segment between caged and uncaged logs. Mean sapstain percentage for *Pinus radiata* log segments (A–F), separated by section along the length of the log for both caged and uncaged logs. Error bars are ± one standard error of the mean.

The amount of sapstain cover varied from as low as 0% in the central section cut and upper segments of the log, to as high as 100% in the terminal cuts and lower segments of the log nearest to the ground (Figure 3A, B). Evidence of sapstain fungal colonisation was evident to some degree in all logs in the study, whether they were uncaged or caged. However, on average the uncaged logs had a noticeably greater occurrence of stain along the lower segments of the log which were in contact with the ground (Figure 3A, 4E, F), whereas caged logs typically had intense staining only near the terminal (cut) ends of these logs (Figure 3B). Surprisingly, in the Poisson lognormal GLMM analyses this latter effect of varying stain between section cuts of caged versus uncaged logs did not contribute significantly to the best-fit model based on comparison of Akaike weights (Table 1a). Instead, the best-supported model showed only significant effects of caging and segment location (i.e., the radial angle of segments), as well as their associated interaction, on the proportion cover of stain per segment (Table 1a); that is, the distribution of stain among segments varied depending on whether the log was caged or not. The caging by segment interaction effect was driven by higher levels of stain at the bottom of the un-caged logs than at the bottom of the caged logs (Table 2a, Figure 4E, F).

**Table 1:** Results of model selection assessing all combinations of fixed factors using AICc values of the GLMMs.

Model	AICc	ΔAICc	W_m_	Rank
(a) Stain – Poisson lognormal				
cage * segment	6973.9	0.0	1.0	1
segment	7110.9	137.0	0.0	2
cage	7666.8	692.9	0.0	3
cage + section	7671.4	697.5	0.0	4
section	7474.0	700.1	0.0	5
cage * section	7676.0	702.1	0.0	6
cage * section/segment	8257.3	1283.4	0.0	7
section/segment	8334.6	1360.7	0.0	8
cage + section/segment	8341.8	1367.9	0.0	9
cage + segment	40937.9	33960.4	0.0	10
(b) Beetles - binomial				
cage * segment	861.7	0.0	1.0	1
cage + segment	912.2	50.5	0.0	2
segment	946.5	84.8	0.0	3
cage + section/segment	1047.6	185.8	0.0	4
section/segment	1080.7	219.0	0.0	5
cage * section/segment	1449.5	587.8	0.0	6
cage	1496.9	635.2	0.0	7
cage + section	1499.7	638.0	0.0	8
cage * section	1501.4	639.7	0.0	9
section	1531.0	669.3	0.0	10
(c) Other arthropods – binomial				
cage * segment	1230.0	0.0	1.0	1
segment	1270.0	40.0	0.0	2
cage + segment	1270.2	40.2	0.0	3
cage * section/segment	1289.8	59.8	0.0	4
section/segment	1303.9	73.8	0.0	5
cage + section/segment	1304.0	74.0	0.0	6
cage	2194.4	964.4	0.0	7
section	2196.9	966.9	0.0	8
cage + section	2197.8	967.8	0.0	9
cage * section	2207.5	977.5	0.0	10
(d) Stain (with beetle predictor) – Poisson lognormal				
beetles + other arthropods + cage * segment	6973.5	0.0	0.6	1
**beetles + other arthropods + segment**	6974.2	0.7	0.4	2
beetles + other arthropods + cage + segment	6979.4	5.9	0.0	3
beetles + cage + segment	7054.6	81.1	0.0	4
beetles + cage * segment	7057.8	84.3	0.0	5
other arthropods	7183.7	210.1	0.0	6
beetles + other arthropods	7215.1	241.6	0.0	7
beetles + other arthropods + cage	7254.5	281.0	0.0	8
beetles	7356.3	382.8	0.0	9
beetles * cage	7377.1	403.6	0.0	10
beetles * segment	122846.0	115872.5	0.0	11
beetles + cage	207836.0	200862.5	0.0	12

(a) sapstain within *Pinus radiata* logs as a response variable in GLMMs with a Poisson lognormal error structure to account for overdispersion in the binomial stain model, (b) evidence of bark beetle (*Hylastes ater* and *Hylurgus ligniperda*) colonisation as a response in GLMMs with binomial error structure, (c) evidence of other arthropod colonisation as a response in GLMMs with binomial error structure, and (d) GLMMs with Poisson lognormal error structure testing whether the fixed covariate effects of beetle evidence and other-arthropod evidence explain the distribution of stain within and among logs. All models included site replicate and log replicate as random factors. Models within two ΔAICc units of the best model (ΔAICc = 0) were considered to have equivalent explanatory power, as indicated in bold. All models also included the random effects of site replicate and log replicate.

**Table 2:** Coefficients output from selected models showing significance values for all factors.

Fixed factors	Estimate	Standard error	Z value	*P*
(a) Stain – Poisson lognormal				
intercept	0.530	0.654	0.811	0.417
cage	−0.535	0.712	−0.752	0.452
segmentD	−1.940	0.475	−4.089	**<0.001**
segmentB	−3.848	0.507	−7.589	**<0.001**
segmentA	−3.519	0.498	−7.063	**<0.001**
segmentC	−1.213	0.468	−2.592	**0.010**
segmentE	0.701	0.470	1.491	0.136
cage:segmentD	0.724	0.669	1.083	0.279
cage:segmentB	0.990	0.706	1.402	0.161
cage:segmentA	1.597	0.690	2.313	**0.021**
cage:segmentC	1.095	0.660	1.659	0.097
cage:segmentE	0.582	0.663	0.878	0.380
(b) Beetles – binomial				
intercept	2.899	0.619	4.680	**<0.001**
cage	−6.568	0.767	−8.550	**<0.001**
segmentD	−5.389	0.484	−11.140	**<0.001**
segmentB	−6.338	0.567	−11.170	**<0.001**
segmentA	−5.941	0.525	−11.330	**<0.001**
segmentC	−4.759	0.454	−10.480	**<0.001**
segmentE	−0.833	0.428	−1.940	0.052
cage:segmentD	3.738	0.789	4.740	**<0.001**
cage:segmentB	−11.222	1391.715	−0.010	0.994
cage:segmentA	4.292	0.814	5.270	**<0.001**
cage:segmentC	4.443	0.646	6.870	**<0.001**
cage:segmentE	1.962	0.594	3.290	**0.001**
(c) Other arthropods – binomial				
intercept	3.531	0.623	5.671	**<0.001**
cage	−3.020	0.621	−4.866	**<0.001**
segmentD	−5.372	0.531	−10.120	**<0.001**
segmentB	−6.824	0.626	−10.890	**<0.001**
segmentA	−6.430	0.588	−10.93	**<0.001**
segmentC	−4.513	0.510	−8.852	**<0.001**
segmentE	−0.688	0.561	−1.227	0.220
cage:segmentD	3.038	0.618	4.916	**<0.001**
cage:segmentB	2.775	0.775	3.58	**<0.001**
cage:segmentA	2.380	0.745	3.197	**0.001**
cage:segmentC	3.466	0.582	5.955	**<0.001**
cage:segmentE	1.943	0.647	3.002	**0.003**
(d) Stain (beetle & other arthropod predictors) – Poisson lognormal				
intercept	−0.096	0.874	−0.110	0.913
beetles	0.830	0.422	1.970	**0.049**
other arthropods	1.934	0.343	5.640	**<0.001**
cage	−0.336	0.827	−0.410	0.684
segmentD	−1.471	0.572	−2.580	**0.010**
segmentB	−3.539	0.627	−5.650	**<0.001**
segmentA	−3.130	0.610	−5.130	**<0.001**
segmentC	−0.750	0.546	−1.380	0.169
segmentE	0.816	0.479	1.700	0.088
cage:segmentD	0.490	0.725	0.680	0.499
cage:segmentB	0.686	0.778	0.880	0.378
cage:segmentA	1.387	0.759	1.870	0.068
cage:segmentC	0.862	0.714	1.210	0.227
cage:segmentE	0.464	0.677	0.690	0.493

(a) stain in *Pinus radiata* logs as a response variable in a GLMM with a Poisson lognormal error structure, (b) evidence of bark beetles (*Hylastes ater* and *Hylurgus ligniperda*) as a response in a GLMM with binomial error structure, (c) evidence of other arthropods as a response in a GLMM with binomial error structure, and (d) stain as a response variable in a GLMM with a Poisson lognormal error structure, incorporating beetle evidence and arthropod evidence as covariate predictors of stain distribution.

### Spatial distribution of arthropod colonisation

The caged logs were successful at excluding, on average, 93.8% of the beetle colonisation of an uncaged log, with a total of only 26 bark beetles found among all the cages logs, in comparison with 420 in the uncaged logs. Overall, bark beetles penetrated seven (35%) of the caged logs. As expected, the best-fit GLMM model describing bark beetle colonisation included significant effects of the caging treatment and the distribution across segments, as well as their associated interaction (Table 1b). Bark beetle attack was predominantly distributed on the lower surfaces of uncaged logs, and the caging treatment significantly reduced the difference in beetle attack between the upper and lower surfaces of the log (Figure 3C, D). For the few beetles that were able to penetrate the caged logs, colonisation was sporadic but also concentrated in the lower segments of the logs (Figure 3D).

Unavoidably, colonisation by other smaller arthropods was evident in both caged and uncaged logs, at both sites (only 2 out of 20 caged logs had no evidence of arthropod colonisation at all). However, among other arthropods there was no evidence of colonisation by any other adult beetle groups, such as ambrosia beetles. As with bark beetle colonisation, the best-fit GLMM model for other arthropod colonisation also included significant effects of both the caging treatment and segment distribution segments, as well as their associated interaction (Table 1c). Once again, there was a significantly greater level of other arthropod colonisation in the uncaged logs than in the caged logs (Table 2c).

### Arthropod colonisation driving sapstain distribution

Spatial patterns of log colonisation by bark beetles and other arthropods corresponded strongly with sapstain distribution and intensity (Figure 3). A GLMM analysis of stain distribution showed that when variables representing colonisation by beetles and other arthropods were entered first into the model, ahead of caging and segment effects, there was still a highly significant effect of segment position on stain intensity, but only a weak and equivocal remaining influence of the caging effect (Figure 3, Table 1d, Table 2d). This suggests that the caging effect on stain intensity is predominantly driven by reduction in beetle and other arthropod colonisation (despite the fact that some arthropods did penetrate cages), but that the beetle and other arthropod evidence is not sufficient in its own right to explain variation in stain intensity among segments within logs (Table 1d).

### Fungal species causing sapstain

In this study, 10 isolations were attempted from two discs from each of three additional cut trees at the two sites, using ophiostomatoid- and non-ophiostomatoid-selective agar media, giving a total of 120 isolation attempts. There were 26 positive isolations of stain fungi, 100% of which were *Diplodia pinea*, while the majority of other attempted isolations did not produce fungal growth, or consisted of non-staining fungi or bacterial contaminants. No ophiostomatoid fungi were present. The *D. pinea* isolates were identified from both the exposed end of the log (8 isolates) and deep within the log at 4.5 m up the length of the tree (18 isolates).

## Discussion

Sapstain developed and spread in all logs in this study regardless of the presence or absence of potential bark beetle vectors, but beetle colonisation was clearly an important factor in the intensity of fungal colonisation and degree of spread of fungi in cut logs. Our experimental design used a caging treatment to exclude beetle colonisation while keeping other site-level and seasonal environmental factors constant. Although the cages were only 93.8% effective in preventing beetle colonisation, there was nevertheless a strong correspondence between the spatial distributions of bark beetle colonisation and areas of high sapstain intensity within and between logs. Equally, however, it is clear that the feeding and tunnelling activity of other saproxylic arthropods and their larvae also contributed significantly to the onset and extent of stain within timber [23], [42]. Consequently, although bark beetles do appear to be instrumental in the colonisation and spread of sapstain to damaged timber [10], [43], it seems that endophytic or wind blown fungal spores (such as those of *Diplodia pinea*) and the distribution of arthropod activity within fallen timber also play an important role in sapstain spread. Below, we discuss the implications of these findings for our understanding of vector-pathogen dynamics, and for the forest industry to highlight the importance of protecting harvested logs and trees damaged by stochastic climate events such as storm and fire damage.

### Stain and beetle colonisation

There was a low level of stain in the upper segments of all logs, especially toward the middle of the log where the sapwood was protected by the bark, and beetles had not colonised. If left in situ for a longer period of time these sections would presumably also have become stained as the moisture content of the logs declined further, and the ‘clean’ timber succumbed to stain fungi growing from the sites of beetle colonisation and from the exposed ends of the logs. In the uncaged logs, the greater intensity of staining was along the lower surface of the log, where the log was in contact with the ground. This was mirrored almost exactly by the pattern of bark beetle colonisation, in this case by *Hylastes ater* and *Hylurgus ligniperda*. As these species are known to feed preferentially on roots, the lower stem and other parts of trees that are in ground contact, they are behaviourally adapted to preferentially colonise logs where they are in contact with the ground [34].

Although the cages were not entirely effective, the spatial distribution of stain within caged logs was significantly different to that of uncaged logs. Sapstain distribution within caged logs was concentrated at the exposed ends where wind and rain-splash might act as sources of spore inoculum into the unprotected sapwood, and where the sapwood would also dry faster allowing ideal conditions for aerobic growth. In fact, fungal isolations taken from whole cut logs that had previously been felled at each site revealed that the majority of sapstain was caused by the abiotically-dispersed *D. pinea*. Although it is likely that other sapstain fungal species also occur at these sites (e.g. *Ophiostoma* spp.), these must have occurred at such low abundances that they were not detected in this study.

In previous large-scale studies using similar isolation methods, 12 species of ophiostomatoid fungi were identified from *Pinus radiata* timber and from bark beetle vectors [15], [44]. Furthermore, from a recent broad-scale sapstain fungal isolation effort in the same region, with a total of 680 sapstain fungal isolations from *P. radiata* sapwood, 666 were *D. pinea* (97.9%), and the remaining 14 were distributed among *Sporothrix inflata* de Hoog and nine different *Ophiostoma* species (McCarthy et al. unpublished data). This reinforces earlier research identifying *D. pinea* as New Zealand's most widespread and dominant sapstain fungus on pine [15], [16]. Because damaged and diseased plant tissue (including needles and cones on the forest floor) can be a prolific source of *D. pinea* inoculum [20], [45], [46], abiotically-dispersed spores are likely to be ubiquitous within pine forests in New Zealand.

In addition to abiotic spore dispersal, the possibility of endophytic sapstain development must be considered, as some staining species such as *D. pinea* can (unlike ophiostomatoid fungi) be latent endophytes in healthy *Pinus* trees. Among these species, visible evidence of stain development often only becomes evident once a tree has been damaged or stressed [17], [47], such as at the cut ends of the experimental logs. It is unknown whether the stain development in these logs was dominated by endophytic growth or spore inoculation following the cutting of the logs. Certainly, stain development in the middle sections of the experimental logs was dramatically lower where protection by the bark prevented the entry of fungal spores, suggesting spore dispersal and their ability to colonise the sapwood, not endophytic growth, is the major factor to consider.

Given these findings, it appears more likely that the strong correspondence between sapstain intensity and beetle colonisation intensity was due to facilitation of *D. pinea* spore entry through the bark into beetle tunnels, and hyphal development through the network of larval feeding galleries under the bark, potentially from fungal inoculum that predominantly arrived by wind or rain-splash, rather than by beetle vectors. Likewise, the same is true for the colonisation activities of other arthropods.

### Stain and colonisation by other arthropods

In addition to beetle-mediated stain development, there was also a significant influence of the distribution of other (unidentified) arthropod larvae on stain development, in both the caged and uncaged logs. These larvae were likely to have entered the cages through the mesh directly, or been introduced by adult arthropods which burrowed below the log and oviposited through the mesh. The ability of flies, mites, beetles, and their larvae, to vector sapstain fungi has been documented [23], [42], [48], but the possibility of arthropods other than bark beetles as facilitators of sapstain development is not commonly considered. Further research is needed to examine and identify the species involved, and quantify their respective importance as vectors of sapstain fungi, or their role in facilitating fungal infection of host tissues through feeding induced tree-wounds. There is evidence of *D. pinea* exploiting the entry points of wood created by arthropods including a variety of bark beetles and wood borers [22], [49], as well as other taxa such as the aphid *Cinara cronartii* T&P [22], the scale insect *Matsucoccus josephi* Bodenheimer and Harpaz [50], the moth *Dioryctria* sp. [51], and the true bug *Leptoglossus occidentalis* Heidemann [52].

### Applied management implications

The risk of timber discolouration due to sapstain colonisation is greatest during harvesting, storage and transport of logs, and following storm events where salvage-harvesting of damaged trees may be an economically viable option. Bark beetles and other arthropods play an important role in the facilitation of fungal entry into susceptible logs, and protection of logs from these organisms should be attempted. This study shows that if logs are not protected from arthropod colonisation then sapstain will progress rapidly, acting either additively or—most likely—synergistically with wind-blown spores to increase the distribution and intensity of staining in timber. In quantitative terms, it should be noted that this will happen at a slower rate than observed in our experimental logs because entire fallen trees would retain their moisture for longer, depending on the area of exposed sapwood and environmental conditions at the site.

In the case of storm damaged stands where salvage harvest may be warranted, New Zealand is in the situation where the two major bark beetles species, *H. ater* and *H. ligniperda*, are behaviourally adapted to feed preferentially on roots and other parts of the tree that are in direct contact with the ground [34]. Following storm events, the fallen stems of most trees are often elevated above ground-level by their branches, and this limits the amount of the log likely to be colonised. In a recent study investigating the onset of sapstain following windthrow, only 28 of 480 discs (5.8%) sampled systematically from fallen trees had any form of contact with the ground or another tree (McCarthy et al., unpublished data). Although these trees are likely to sustain some beetle colonisation, and are susceptible to staining by wind-dispersed and endophytic fungi [15], the extent of damage will probably be lessened in the absence of extensive beetle colonisation. Mausel et al. [34] found that logs stacked in a manner that reduced contact with the ground, or with other logs, greatly limited colonisation by both *H. ater* and *H. ligniperda*. This is likely to be the case for other bark beetle species with similar colonisation strategies, where colonisation of stacked logs could be reduced with the use of “spacers”, or similar, to reduce the points of contact between stacked logs, as this is when bark beetle colonisation would be likely. In other regions of the world, effective protection of susceptible logs, whether they be harvested or storm damaged, will require examination of the bark beetle species most likely to colonise, and their colonisation strategies. In areas with more aggressive bark beetles that colonise logs under all conditions, more intensive methods of arthropod and fungal management will be required or very rapid log processing to avoid sapstain of commercial timber.
